# Optimization of direct oral anticoagulant prescribing in primary care: a pharmacotherapeutic review in real-world clinical practice and therapeutic efficiency

**DOI:** 10.3389/fphar.2026.1969085

**Published:** 2026-10-14

**Authors:** Núria Gotanegra Guzmán, Marta Turu Pedrola, Montserrat Viñas-Bastart, Carlos Alonso-Villaverde Lozano, Irene Teixidó Javierre, Mercè Medina Vargas, Pilar Modamio, Josep Maria Guiu-Segura

**Affiliations:** 1 Pharmacy Service, Xarxa Tecla Sanitaria, Social I Docent, Tarragona, Spain; 2 Pharmacy Unit, Camp de Tarragona Health Region, Catalan Health Service (CatSalut), Tarragona, Spain; 3 Clinical Pharmacy and Pharmaceutical Care Unit, Department of Pharmacy and Pharmaceutical Technology, and Physical Chemistry, Faculty of Pharmacy and Food Sciences, University of Barcelona, Barcelona, Spain; 4 Clinical Research Unit, Xarxa Tecla Sanitaria, Social I Docent, Tarragona, Spain

**Keywords:** dabigatran, direct oral anticoagulants, healthcare efficiency, medication optimization, pharmacist-led intervention, prescribing appropriateness, primary care

## Abstract

**Introduction:**

Direct oral anticoagulants are among the first-line treatments for stroke prevention in patients with non-valvular atrial fibrillation and for the treatment and secondary prevention of venous thromboembolism. The increasing use of these agents poses challenges related to prescribing appropriateness, clinical safety. This study aims to evaluate the impact of a primary care pharmacist-led intervention on the appropriateness of DOAC prescribing, pharmacotherapeutic efficiency, and clinical outcomes.

**Materials and Methods:**

A retrospective, quasi-experimental before-and-after study was conducted in the healthcare area of Xarxa Santa Tecla (Tarragonès and Baix Penedès, Spain). 791 patients receiving DOAC therapy were included. The intervention comprised multidisciplinary educational activities, individualized pharmacotherapeutic review, and communication of recommendations to prescribers.

**Results:**

The mean age was 78.2 years, and 43.2% of patients had moderate or severe renal impairment. The pharmacotherapeutic review identified 527 patients (66.6%) as candidates for treatment optimization, through a proposed switch from apixaban to dabigatran. The pharmacist intervention was associated with changes in prescribing patterns among newly initiated patients, increasing the use of dabigatran. The economic analysis estimated savings of €75,508.81 from new treatment initiations and €31,288.25 from therapeutic switches, resulting in total savings of €106,797.06 in 2025. The overall bleeding rate remained stable (5.3% in 2024% vs. 5.2% in 2025), with no deterioration in clinical safety. In conclusion, a structured, multidisciplinary, and coordinated primary care pharmacist-led intervention promoted a more efficient use of healthcare resources, while no deterioration in population-level safety indicators was observed. These findings reinforce the role of primary care pharmacists in optimizing anticoagulant therapy.

## Highlights

- Pharmacist-led review improved DOAC prescribing in primary care.
- Dabigatran prescribing increased after the intervention.
- Recommendations targeted inappropriate DOAC use in clinical practice.
- The intervention generated substantial healthcare cost savings.
- Findings support pharmacist-led pharmacotherapeutic optimization.

## 1 Introduction

Thromboembolic disease is one of the leading cardiovascular health problems worldwide due to its high morbidity, mortality, and healthcare burden. Within this spectrum, non-valvular atrial fibrillation (NVAF) and venous thromboembolism (VTE), which includes deep vein thrombosis (DVT) and pulmonary embolism (PE), represent two of the main indications for oral anticoagulant therapy in routine clinical practice (Programa d’Harmonització Farmacoterapèutica PHF, 2025; Agencia Española de Medicamentos y Productos Sanitarios AEMPS, 2024). Both conditions are associated with a substantial risk of thromboembolic complications and frequently require long-term anticoagulant treatment to prevent recurrence, embolic events, and associated mortality (Programa d’Harmonització Farmacoterapèutica PHF, 2025; Agencia Española de Medicamentos y Productos Sanitarios AEMPS, 2024).

Anticoagulation is the cornerstone of treatment for both NVAF and VTE. In patients with NVAF, the prevention of stroke and systemic embolism is one of the main therapeutic goals, whereas in patients with VTE, anticoagulant therapy reduces thrombus progression, recurrence, and disease-related mortality (Agencia Española de Medicamentos y Productos Sanitarios AEMPS, 2024; Ageno et al., 2024). Traditionally, anticoagulation has relied on vitamin K antagonists (VKAs), mainly acenocoumarol and warfarin, together with the use of heparins during the initial treatment phase of VTE (Programa d’Harmonització Farmacoterapèutica PHF, 2025; Agencia Española de Medicamentos y Productos Sanitarios AEMPS, 2024). However, VKAs have several important limitations, including a narrow therapeutic window, considerable interindividual variability, numerous drug and food interactions, and the need for regular monitoring using the international normalized ratio (INR) (Agencia Española de Medicamentos y Productos Sanitarios AEMPS, 2024).

The introduction of direct oral anticoagulants (DOACs) has represented one of the most significant therapeutic advances in anticoagulation over the past 2 decades (Ageno et al., 2024; Grymonprez et al., 2022). Dabigatran etexilate was the first DOAC to become available, initially approved in Europe in 2008 for the prevention of venous thromboembolism following orthopedic surgery and subsequently for the prevention of stroke and systemic embolism in patients with NVAF. It was followed by the direct factor Xa inhibitors rivaroxaban, apixaban, and edoxaban, thereby expanding the therapeutic options available for the prevention and treatment of thromboembolic disease (Agencia Española de Medicamentos y Productos Sanitarios AEMPS, 2024).

Pivotal clinical trials and real-world studies have demonstrated efficacy, effectiveness and safety profiles that are at least comparable to those of VKAs, together with a consistent reduction in the risk of intracranial hemorrhage, fewer drug interactions, and no requirement for routine INR monitoring (Ageno et al., 2024; Grymonprez et al., 2022; Connolly et al., 2009; Alshahrani et al., 2024; Coyle et al., 2024). These advantages have simplified anticoagulant therapy and facilitated its management in routine clinical practice (Agencia Española de Medicamentos y Productos Sanitarios AEMPS, 2024; Patrice et al., 2024). Consequently, current clinical practice guidelines recommend DOACs as first-line therapy for stroke and systemic embolism prevention in patients with NVAF. In patients with VTE, both DOACs and VKAs are considered effective and safe therapeutic options, and treatment selection should be individualized according to each patient’s clinical characteristics (Patrice et al., 2024; Agencia Española de Medicamentos y Productos Sanitarios AEMPS, 2024).

For many years, the selection among different DOACs was primarily based on clinical considerations such as therapeutic indication, renal function, bleeding risk, drug interactions, and individual patient characteristics. However, the introduction of the first generic medicine of dabigatran in Spain in August 2022 changed this scenario (Agencia Española de Medicamentos y Productos Sanitarios, 2026a). The first reimbursed generic medicines were incorporated into the Spanish National Health System formulary in December 2023, resulting in a substantial reduction in treatment costs compared with the other available DOACs (Ministerio de Sanidad, 2024).

Nevertheless, current clinical guidelines do not establish the superiority of one DOAC over another. Therefore, treatment selection should be individualized by considering clinical and pharmacological factors including renal and hepatic function, bleeding risk, age, body weight, concomitant medications, treatment adherence, and patient preferences (Patrice et al., 2024; Agencia Española de Medicamentos y Productos Sanitarios AEMPS, 2024; Parks et al., 2024). Certain clinical situations may also favor the selection of a specific DOAC, particularly in elderly, frail, or multimorbid patients and in those with renal impairment (Parks et al., 2024; Menichelli et al., 2025). In this context, efficiency criteria may be incorporated into therapeutic decision-making only when different treatment options are considered clinically appropriate for the same patient.

Among the available DOACs, apixaban and dabigatran are two of the most widely prescribed agents in clinical practice. Both have demonstrated efficacy comparable to that of warfarin, together with favorable safety profiles (Coyle et al., 2024; Van Ganse et al., 2020). Nevertheless, they differ in several clinically relevant aspects that may influence treatment selection. Dabigatran requires at least 5 days of prior parenteral anticoagulation for the initial treatment of VTE and is predominantly eliminated through the kidneys, whereas apixaban can be initiated without prior parenteral anticoagulation and is less dependent on renal function (Programa d’Harmonització Farmacoterapèutica PHF, 2025; Parks et al., 2024; Menichelli et al., 2025). Consequently, careful assessment of renal function is essential when selecting dabigatran, and treatment choice should always be individualized according to the patient’s clinical characteristics and approved therapeutic indications**.**

The safe use of DOACs requires structured clinical follow-up. Several consensus documents have highlighted that anticoagulation-related medication errors may occur during prescribing, transitions of care, switching between anticoagulants, or treatment discontinuation (Programa d’Harmonització Farmacoterapèutica PHF, 2025; Agencia Española de Medicamentos y Productos Sanitarios AEMPS, 2024). The most common medication-related problems include inappropriate dosing, unrecognized drug interactions, therapeutic duplication, and poor treatment adherence (Programa d’Harmonització Farmacoterapèutica PHF, 2025; Chang et al., 2017). Consequently, regular reassessment of renal and hepatic function, bleeding risk, and the overall appropriateness of anticoagulant therapy is recommended (Joglar et al., 2023; Steffel et al., 2021).

The progressive increase in DOAC use over the last decade has also generated a considerable economic impact on healthcare systems (Niyomsri et al., 2023; Sun et al., 2021). Although these therapies may be cost-effective by reducing major thromboembolic and hemorrhagic events, their acquisition costs remain higher than those of VKAs (Niyomsri et al., 2023; Sun et al., 2021).

Against this background, primary care pharmacists play a key role in optimizing anticoagulant therapy. Structured pharmacist-led interventions have been shown to improve prescribing appropriateness, identify medication-related problems, and support appropriate anticoagulant selection according to the best available evidence (Chambers et al., 2024; Rankin et al., 2018). Furthermore, collaboration between pharmacists and physicians promotes individualized therapeutic decision-making by integrating clinical variables, patient preferences, and efficiency criteria (Programa d’Harmonització Farmacoterapèutica PHF, 2025; Agencia Española de Medicamentos y Productos Sanitarios AEMPS, 2024).

In this context the present study aimed to evaluate the impact of a primary care pharmacist-led intervention on the appropriateness of DOAC prescribing. In addition, the study sought to identify clinical and pharmacotherapeutic factors associated with prescribing appropriateness, assess the acceptance of pharmacists’ recommendations, estimate the economic impact of the intervention, and evaluate the persistence of therapeutic changes over time.

## 2 Materials and methods

### 2.1 Study design

This was a retrospective, quasi-experimental before-and-after study based on a pharmacist-led medication review and optimization program integrated into routine quality improvement activities conducted by the primary care pharmacy service. The study was carried out between June and December 2025 and involved neither experimental treatment allocation nor protocol-driven modifications to routine clinical practice.

### 2.2 Study setting

The study was conducted within the healthcare network of Xarxa Santa Tecla Sanitària, Social i Docent (Tarragonès and Baix Penedès, Catalonia, Spain), serving a reference population of 187,717 inhabitants. The healthcare setting comprised six primary care centers (Tarragona IV, Vila-seca, Torredembarra, Calafell, L'Arboç, and El Vendrell), two acute-care hospitals (Hospital de Sant Pau i Santa Tecla and Hospital del Vendrell), two intermediate care facilities (Santa Tecla Llevant and Santa Tecla Ponent), and nineteen nursing homes.

### 2.3 Study population and selection criteria

The study population consisted of adult patients receiving active treatment with apixaban or patients newly initiated on DOAC during the study period.

The sample included the complete census of patients meeting the inclusion criteria in January 2025, comprising a total of 791 patients. This sample size exceeded the minimum number required to detect clinically relevant differences with a 95% confidence level and an estimated precision of 3%–4%.

Eligible patients were adults aged 18 years or older who were receiving active treatment with apixaban or had recently initiated any DOAC during the study period. Patients were excluded if they had documented contraindications to specific DOACs that could influence therapeutic selection, or if they had a history of intolerance or known hypersensitivity to any DOAC.

### 2.4 Description of the pharmacist-led intervention

The intervention was coordinated by the primary care pharmacy unit and implemented at two complementary levels of healthcare. First, newly initiated DOAC prescriptions originating from hospital care—including hospital discharge, outpatient clinics, and emergency departments—were reviewed to promote the selection of the most appropriate anticoagulant according to predefined clinical criteria. Second, patients receiving ongoing apixaban therapy in primary care underwent an individualized medication review to assess the feasibility of therapeutic optimization through switching to dabigatran whenever clinically appropriate.

The intervention was structured into three consecutive phases:

Phase 1. Educational Activities and Interprofessional Coordination.

In January 2025, multidisciplinary educational sessions involving professionals from different healthcare levels were conducted for primary care physicians, hospital specialists, and community pharmacists. Educational materials and specific infographics describing the characteristics and appropriate use of the different DOACs were also developed and distributed.

Participants included specialists in cardiology, internal medicine, hematology, neurology, emergency medicine, family and community medicine, nursing, hospital pharmacy, primary care pharmacy, and community pharmacy.

Phase 2. Individualized Pharmacotherapeutic Review.

During the second phase, a structured individualized pharmacotherapeutic review was conducted for both patients receiving apixaban and treatment-naïve patients eligible for DOAC therapy. The assessment included the clinical indication for anticoagulation, demographic characteristics (age, sex, and body weight), renal function, potential drug–drug interactions, relevant comorbidities, previous allergy or intolerance to dabigatran, the presence of prosthetic heart valves, active cancer, and clinical frailty status, assessed using the Frail-VIG index, a validated frailty assessment tool developed in the Spanish healthcare setting. The index comprises 22 items covering eight dimensions and generates a score ranging from 0 to 1, with higher scores indicating greater frailty. Frailty was categorized as no frailty (<0.20), incipient frailty (0.20–<0.35), intermediate frailty (0.36–0.50), or advanced frailty (>0.50). Based on this comprehensive evaluation, pharmacists provided individualized therapeutic recommendations in accordance with the best available scientific evidence, the approved Summary of Product Characteristics (SmPCs) for each DOAC, and current evidence-based clinical practice guidelines.

Phase 3. Communication of Recommendations and Shared Clinical Decision-Making.

During the third phase, pharmacists communicated their recommendations individually to prescribing physicians. According to each patient’s clinical assessment, the proposed interventions included switching from apixaban to dabigatran, maintaining the current anticoagulant regimen when considered appropriate, or adjusting the anticoagulant dose when clinically indicated. The final therapeutic decision was made by the treating physician.

### 2.5 Outcome variables

For treatment-naïve patients, the primary outcome measures included the total number of patients reviewed, the number and proportion of patients initiated on each DOAC, changes in prescribing patterns following the pharmacist-led intervention, and the estimated pharmaceutical expenditure associated with newly initiated DOAC therapy. For patients receiving apixaban, the primary outcomes comprised the total number of patients reviewed, the number and proportion considered eligible for therapeutic optimization, the number and proportion for whom a therapeutic switch was recommended, the acceptance rate of pharmacists’ recommendations by prescribing physicians, changes in DOAC prescribing patterns, and the evolution of pharmaceutical expenditure associated with DOAC therapy before and after the intervention.

### 2.6 Data sources and variables collected

The data used in this study were extracted from the organization’s electronic health records, electronic prescribing system, and institutional healthcare databases. These sources provided the demographic, clinical, pharmacotherapeutic, intervention-related, and economic data required for the analyses. The appropriateness of DOAC dosing was evaluated according to the European Medicines Agency approved Summary of Product Characteristics (SmPCs) and current clinical practice guidelines. When key clinical information required to assess treatment appropriateness (particularly body weight or renal function) was unavailable, no imputation of missing data was performed. Such patients remained in the descriptive analyses; however, no pharmacotherapeutic recommendation was issued until the required information became available.

All analyses were performed using anonymized data extracted from the institutional clinical information systems.

### 2.7 Statistical analysis

A descriptive analysis of the study variables was performed. Continuous variables were expressed as mean ± standard deviation (SD) or median and interquartile range (IQR), depending on their distribution. Categorical variables were summarized as absolute frequencies and percentages. To evaluate the impact of the strategy, prescribing patterns and estimated pharmaceutical costs before and after the pharmacist-led intervention were compared. Results were expressed as percentages, absolute differences, and estimated cumulative cost savings.

### 2.8 Ethical considerations

The study protocol was reviewed and approved by the Clinical Research Ethics Committee (CEIm) of IISPV (Institut d’Investigació Sanitària Pere Virgili) on 23 July 2026 (approval No. 144/2026). The CEIm granted an exemption from the requirement for individual informed consent, as documented in the ethics committee approval.

As this retrospective study evaluated outcomes obtained from routine clinical practice without modifying standard patient care, the study was conducted without individual informed consent. The study was conducted in accordance with the principles of the Declaration of Helsinki. Patient confidentiality was ensured through anonymization and data de-identification procedures in compliance with applicable data protection legislation.

## 3 Results

### 3.1 Baseline characteristics of the study population

Two patient groups corresponding to the two intervention strategies were initially included.

The first cohort consisted of treatment-naïve patients who initiated DOAC therapy between January and December 2025 across the different healthcare settings involved in the intervention, including outpatient clinics, hospital discharge units, and emergency departments. This cohort comprised 1,234 patients, of whom 532 (43.1%) initiated dabigatran, 361 (29.3%) apixaban, 214 (17.3%) edoxaban, and 127 (10.3%) rivaroxaban. Following the pharmacist-led intervention, prescribing patterns shifted towards greater use of dabigatran as the preferred first-line DOAC when clinically appropriate. From January to December 2025, the number of treatment-naïve patients initiating dabigatran increased by 72.4% (from 29 to 50 initiations per month), whereas initiations of the other DOACs declined over the same period: rivaroxaban by 44.4% (18–10), apixaban by 24.2% (33–25), and edoxaban by 64.5% (31–11).

The second cohort included a total of 791 patients receiving apixaban from six primary care centers (Table 1). The study population was distributed relatively evenly across the participating centers. The mean age of the cohort was 78.2 years, and 55.0% were male, which is consistent with the epidemiological profile reported for patients with atrial fibrillation in routine clinical practice (Van Ganse et al., 2020; Bhandari et al., 2025). This elderly population is particularly relevant, as advanced age is associated with greater clinical complexity, polypharmacy, and an increased risk of both thromboembolic and bleeding events (Van Ganse et al., 2020; Bhandari et al., 2025).

**TABLE 1:** Baseline characteristics of the study population (*n* = 791).

Variable	n (%)
Age (years, mean ± SD)	78.2 ± 9.94
Sex	
Female	356 (45.0)
Male	435 (55.0)
Body weight (kg)	78.2
Not recorded	301 (38.1)
Estimated glomerular filtration rate (eGFR) (mL/min)	
>90	58 (7.3)
60–89	334 (42.2)
15–59	341 (43.2)
<15	1 (0.1)
Not recorded	57 (7.2)
GFR	
(>90 mL/min	58 (7.3)
60–89 mL/min	334 (42.2)
30–44 mL/min	132 (16.8)
45–59 mL/min	171 (21.6)
16–29 mL/min	38 (4.8)
<15 mL/min	1 (0.1)
Not recorded	57 (7.2)
Relevant medical history	
No relevant medical history	657 (83.1)
Active cancer	104 (13.2)
Prosthetic heart valve	8 (1.0)
Other	22 (2.8)
Frailty status (Frail-VIG)	
Missing data	615 (77.8)
No frailty	16 (2.0)
Incipient frailty	54 (6.8)
Intermediate frailty	73 (9.2)
Advanced frailty	33 (4.2)

Regarding renal function, more than 85% of patients had an estimated glomerular filtration rate (eGFR) between 15 and 89 mL/min, while 43.2% had an estimated eGFR <60 mL/min/1.73 m^2^, corresponding to a level of renal function compatible with CKD, stage 3 or higher, although chronic kidney disease could not be confirmed because longitudinal data were unavailable. Furthermore, renal function data were unavailable for 7.2% of patients.

From a clinical perspective, most patients had no particularly relevant medical history affecting anticoagulant selection. However, 13.2% had active cancer, and a small proportion had prosthetic heart valves, a clinical scenario in which DOAC therapy may not be appropriate.

Regarding frailty, frailty status was available for 176 patients (22.2%). Among these, 16 patients (2.0% of the total cohort) had no frailty, while 54 (6.8%) had incipient frailty, 73 (9.2%) had intermediate frailty, and 33 (4.2%) had advanced frailty.

### 3.2 Clinical status and factors related to anticoagulation safety

Regarding medication safety, most patients (88.1%) had no clinically relevant drug-drug interactions, whereas 11.9% presented at least one potential interaction, with amiodarone being the most frequently involved medication (Table 2).

**TABLE 2:** Potential drug-drug interactions identified in the study population.

Variable	*n* (%)
No drug-drug interactions	697 (88.1)
One potential interaction	91 (11.5)
Two potential interactions	3 (0.4)
Drugs responsible for the drug–drug interaction	
Amiodarone	65 (8.2)
Diltiazem	12 (1.5)
Diltiazem + amiodarone	2 (0.3)
Dronedarone	2 (0.3)
Spironolactone	9 (1.1)
Other drugs	<1% each

### 3.3 Transition from vitamin K antagonists to direct oral anticoagulants (2018–2025)

To provide context for the pharmacist-led intervention, the annual trends in the number of patients receiving oral anticoagulant therapy between 2018 and 2025 were analyzed (Figure 1).

**FIGURE 1:**
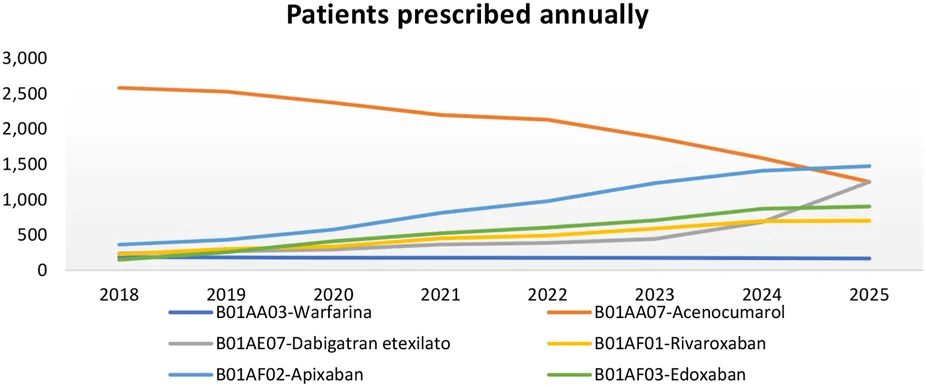
Annual trends in the number of patients receiving vitamin K antagonists (VKAs) and direct oral anticoagulants (DOACs) in the Xarxa Santa Tecla healthcare area (2018–2025).

During the study period, the total number of anticoagulated patients increased progressively from 3,743 in 2018 to 5,754 in 2025, representing an absolute increase of 2,011 patients (+53.7%). This increase was accompanied by a marked shift in oral anticoagulant prescribing patterns. While the use of VKAs steadily declined, the prescription of DOACs increased continuously throughout the study period.

Specifically, the number of patients receiving acenocoumarol decreased from 2,585 in 2018 to 1,253 in 2025 (−51.5%), whereas warfarin use remained relatively stable, decreasing slightly from 185 to 166 patients (−10.3%).

Conversely, the use of all DOACs increased substantially. Apixaban showed the largest absolute increase, rising from 363 to 1,476 patients (+306.6%), followed by dabigatran (236 vs. 1,252; +430.5%), edoxaban (149 vs. 904; +506.7%), and rivaroxaban (225 vs. 703; +212.4%).

As a consequence, the overall distribution of oral anticoagulant therapy changed considerably. In 2018, VKAs accounted for 74.0% of all anticoagulant treatments, whereas DOACs represented 26.0%. By 2025, this distribution had almost completely reversed, with DOACs accounting for 75.8% of all anticoagulant prescriptions compared with 24.2% for VKAs (Table 3). These findings demonstrate a progressive transition from VKAs to DOACs, which became the predominant class of oral anticoagulants prescribed during the study period.

**TABLE 3:** Evolution of the distribution of patients according to oral anticoagulant class (VKAs vs. DOACs) during the period 2018–2025.

Year	VKAs, n (%)	DOACs, n (%)
2018	2,770 (74.0)	973 (26.0)
2019	2,711 (68.4)	1,255 (31.6)
2020	2,704 (63.6)	1,465 (36.4)
2021	2,353 (54.8)	1,982 (45.2)
2022	2,310 (47.5)	2,466 (52.5)
2023	2,054 (40.8)	2,981 (59.2)
2024	1,761 (32.1)	3,659 (67.9)
2025	1,419 (24.2)	4,335 (75.8)

The values are expressed as the number of patients and the percentage of all anticoagulated patients in each calendar year.

### 3.4 Pharmacist-led recomendations

The pharmacist-led intervention identified a substantial number of patients eligible for therapeutic optimization, confirming considerable room for improvement in prescribing appropriateness. The principal recommendation was to switch treatment to dabigatran, which was proposed for 527 patients (66.6%), the majority of whom were receiving apixaban (*n* = 348; 66%) (Table 4).

**TABLE 4:** Pharmacist-Led recommendations performed.

Type of recommendation	n (%)
Switch to dabigatran	527 (66.6)
No change	216 (27.3)
Apixaban dose reduction	25 (3.2)
Apixaban dose increase	8 (1.0)
Missing data	15 (1.9)

Impact of the Intervention on DOAC Prescribing Patterns.

The pharmacist-led intervention was associated with changes in prescribing patterns among treatment-naïve patients, defined as individuals initiating oral anticoagulant therapy in hospital outpatient clinics, following hospital discharge, or through emergency department care.

During the January–May 2025 pre-intervention period, new DOAC prescriptions were relatively evenly distributed among the available agents. However, during the June–December 2025 intervention period, a marked increase in dabigatran prescribing was observed, accompanied by a substantial reduction in the use of rivaroxaban and, to a lesser extent, apixaban and edoxaban.

- Economic Impact of the Intervention

Overall, 1,234 treatment-naïve patients initiated DOAC therapy during 2025, with dabigatran being the most frequently prescribed agent (*n* = 532), followed by apixaban (*n* = 361), edoxaban (*n* = 214), and rivaroxaban (*n* = 127). The total actual pharmaceutical expenditure for these treatments amounted to €686,499.85.

When this scenario was compared with a counterfactual model that extrapolated prescribing patterns observed during the pre-intervention period (January–May 2025), assuming that these patterns would have remained relatively stable in the absence of the intervention, the estimated total expenditure was €762,008.66. Under this assumption, the observed prescribing patterns during the intervention period were associated with estimated savings of €75,508.81 among treatment-naïve patients. Among patients for whom a switch from apixaban to dabigatran was recommended, 146 patients (27.7%) were successfully switched to dabigatran in February 2025. This finding is particularly relevant from an efficiency perspective, as dabigatran is substantially less expensive than the other available DOACs in Spain while maintaining comparable efficacy and safety in appropriately selected patients. Based on the average annual treatment cost per patient, these therapeutic switches resulted in estimated annual savings of €62,576.50. Because the intervention was implemented during the second half of the year, the actual savings achieved in 2025 were estimated at €31,288.25.

Overall, the combined impact of changes in treatment initiation and therapeutic switching resulted in estimated pharmaceutical cost savings of €106,797.06 during 2025. These estimates reflect differences in drug acquisition costs only and do not include the costs associated with implementing the pharmacist-led intervention, such as pharmacist time, educational activities, coordination, or the development of supporting materials. Therefore, the reported savings should be interpreted as pharmaceutical expenditure savings rather than total healthcare system savings. The annual drug acquisition cost per patient according to the prescribed DOAC is presented in Table 5.

**TABLE 5:** Annual cost per patient according to DOAC.

DOAC	Annual cost per patient (€)	Difference vs. Dabigatran (€)
Dabigatran etexilate	256.57	—
Apixaban	720.28	+463.71
Rivaroxaban	783.68	+527.11
Edoxaban	767.10	+510.53

- Persistence of Therapeutic Changes at 12-Month Follow-up

Among patients for whom a switch from apixaban to dabigatran was recommended, treatment persistence was evaluated at 10 months through review of active electronic prescriptions (Table 6).

**TABLE 6:** Persistence of dabigatran therapy during follow-up.

Status at 10 months	*n*	%
Continued dabigatran	137	93.8
Switched back to apixaban	5	3.4
Switched to another DOAC	1	0.7
Anticoagulant therapy discontinued	3	2.1

Acceptance of the pharmacist’s recommendation represented the first indicator of successful implementation. Among patients whose recommendation was accepted, continuity of dabigatran therapy and subsequent treatment changes were assessed.

Among the five patients who switched back to apixaban, three experienced adverse events (two gastrointestinal adverse effects and one due to perceived atherothrombotic risk), whereas no clear reason for the switch was documented in the remaining two cases. One patient was switched to another DOAC to improve treatment adherence. Anticoagulant therapy was discontinued in three patients, including one because of advanced frailty and two for reasons pending clinical review.

- Evolution of Thromboembolic and Bleeding Events Before and After the Intervention: Safety and Clinical Effectiveness of the Pharmacotherapeutic Optimization Strategy

Clinical events recorded among hospitalized anticoagulated patients were compared between the January–December 2024 and January–December 2025 periods to explore changes in safety and effectiveness indicators following implementation of the pharmacotherapeutic optimization strategy.

The total number of hospitalizations involving anticoagulated patients increased from 1,132 in 2024 to 1,241 in 2025 (+9.6%), reflecting increased healthcare activity within this population. Despite this increase, the overall bleeding rate remained stable (5.3% vs. 5.2%), suggesting that the intervention was not associated with an increased risk of bleeding.

Regarding thromboembolic outcomes, the absolute number of ischemic strokes increased from 19 to 26 cases, while transient ischemic attacks increased from 3 to 12 cases. However, these events remained infrequent relative to the overall cohort and should therefore be interpreted cautiously.

With respect to bleeding complications, the number of gastrointestinal bleeding episodes remained essentially unchanged (25 vs. 24 cases), whereas hemorrhagic stroke increased from 4 to 8 cases. Although this represented a relatively large proportional increase, the small absolute number of events limits definitive interpretation.

In-hospital mortality increased from 55 to 85 deaths, corresponding to an increase from 4.9% to 6.8% (RR 1.41). However, this finding should be interpreted within the broader temporal context of hospital activity. A complementary analysis including all hospital admissions in the same healthcare area demonstrated that overall in-hospital mortality actually declined between 2024 and 2025 (3.61% vs. 3.41%), indicating that the increase observed among anticoagulated patients did not reflect a general deterioration in hospital outcomes. Moreover, multivariable analyses identified a significant interaction between study year and anticoagulation status (adjusted interaction OR 1.89, 95% CI 1.29–2.78; p = 0.001), showing that mortality trends differed significantly between anticoagulated and non-anticoagulated patients. Within the anticoagulated subgroup, the adjusted odds of in-hospital death were higher in 2025 (adjusted OR 1.43, 95% CI 1.00–2.03; p = 0.049). Decomposition analyses further suggested that most of the excess mortality reflected an increase in case-fatality rather than the higher number of anticoagulated admissions. Importantly, bleeding-related deaths remained essentially unchanged, whereas excess mortality was predominantly observed among patients with acute kidney injury and cardiorespiratory comorbidity. These trends are illustrated in Figure 2, which shows the increase in in-hospital mortality among anticoagulated patients between the two periods, and in Figure 3, which summarizes the corresponding thromboembolic and hemorrhagic events.

**FIGURE 2:**
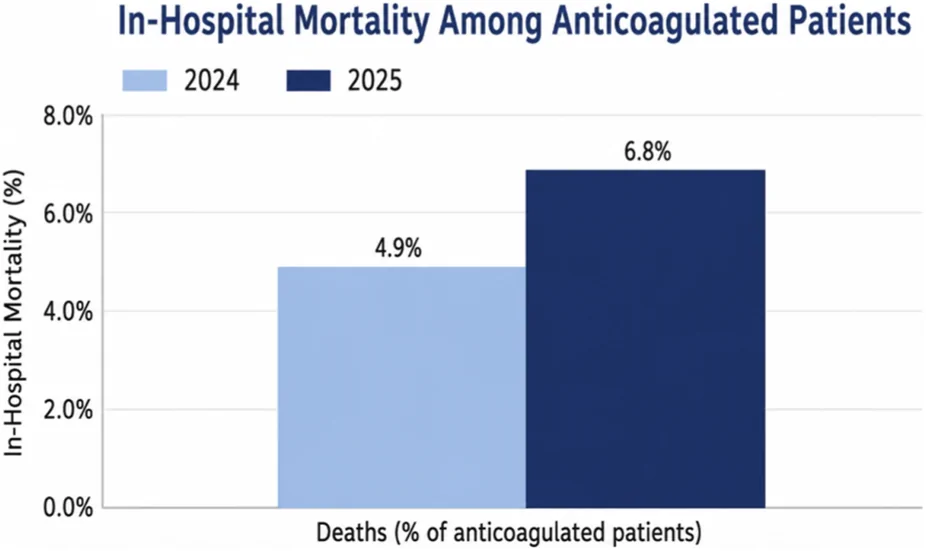
In-hospital mortality in the subcohort of anticoagulated patients during the 2024 and 2025 study periods.

**FIGURE 3:**
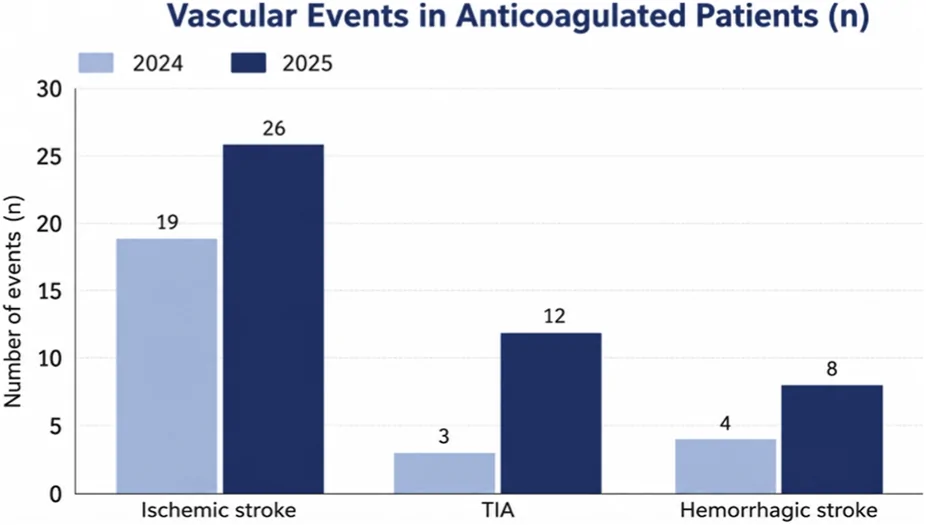
Vascular events in anticoagulated patients (*n*).

Table 7 presents the complete comparison of clinical event rates, relative risks, and statistical significance for each outcome across the two periods.

**TABLE 7:** Comparison of clinical events during the pre-intervention and post-intervention periods. Relative risk (RR) was calculated using the total number of anticoagulated patients in each year as the denominator.

Event	2024 (n = 1,132)	2025 (n = 1,241)	Rate 2024 (%)	Rate 2025 (%)	Relative risk (RR)	P Value
Overall bleeding	60	65	5.30	5.24	0.99	1,000
Ischemic stroke	19	26	1.68	2.10	1.25	0.547
Transient ischemic attack	3	12	0.27	0.97	3.65	0.037
Hemorrhagic stroke	4	8	0.35	0.64	1.83	0.393
In-hospital mortality	55	85	4.86	6.85	1.41	0.045

It should be noted that these data were derived from administrative databases based on ICD-10 coding, and that anticoagulated patients were identified using the Z79.01 code, which does not distinguish between patients receiving VKAs and those receiving DOACs. Consequently, these findings should be interpreted as population-level safety indicators for the overall anticoagulated cohort rather than as a specific evaluation of patients directly exposed to the pharmacist-led DOAC optimization intervention.

## 4 Discussion

Regarding renal function, the high prevalence of renal impairment observed in this cohort highlights an important gap in clinical care, particularly considering that renal function is a key determinant of both DOAC selection and dose adjustment. Current clinical guidelines recommend regular assessment of renal function, especially in older adults, to minimize preventable risks such as overdosing or reduced therapeutic effectiveness (Joglar et al., 2023; Steffel et al., 2021; Tran et al., 2025). In this regard, the pharmacist-led interventions identified patients without recent laboratory monitoring, reinforcing its value as a structured strategy for identifying medication-related risks.

Regarding comorbidities, the presence of active cancer and, less frequently, prosthetic heart valves among a subset of patients suggests the presence of potentially inappropriate anticoagulant prescribing in routine clinical practice, as previously reported in the literature, and reinforce the need for systematic anticoagulation therapy reviews (Steffel et al., 2021).

Regarding frailty, the relatively high prevalence of intermediate and advanced frailty among patients with available Frail-VIG assessment highlights the importance of individualized pharmacotherapeutic optimization strategies in patients with frailty, particularly in the context of multimorbidity (Table 1).

Regarding drug-drug interactions, the potential interactions identified in this cohort, most frequently involving amiodarone, are consistent with the available evidence indicating that, although DOACs have fewer drug interactions than VKAs, clinically relevant interactions mediated by P-glycoprotein (P-gp) and cytochrome P450 3A4 (CYP3A4) still occur and may affect plasma drug concentrations (Steffel et al., 2021).

The present findings should be interpreted within the context of the profound transformation in oral anticoagulant therapy over the past decade. Population-based data from our healthcare area demonstrate a progressive replacement of VKAs by DOACs, which increased from representing approximately one-quarter of all anticoagulant treatments in 2018 to more than three-quarters in 2025. This trend is consistent with reports from Spain and other European countries, where the introduction of DOACs has substantially changed clinical practice owing to their favorable benefit-risk profile and simplified therapeutic management (Ageno et al., 2024; Grymonprez et al., 2022; Joglar et al., 2023).

Within this transition, the marked increase in apixaban use during the early years of the study period is noteworthy, followed by a substantial increase in dabigatran prescriptions after the availability of generic formulations (Programa d’Harmonització Farmacoterapèutica PHF, 2025; Agencia Española de Medicamentos y Productos Sanitarios, 2026b; Ministerio de Sanidad, 2024). This pattern is consistent with the incorporation of efficiency criteria into therapeutic decision-making when different DOACs offer comparable efficacy and safety profiles in appropriately selected patients (Niyomsri et al., 2023; Sun et al., 2021; Godman et al., 2014).

Although the gradual replacement of VKAs by DOACs reflects a broader prescribing trend independent of the present strategy, the pharmacist-led strategy helped guide this transition toward clinically appropriate and more cost-efficient alternatives, thereby promoting a more rational use of healthcare resources without compromising patient safety (Chambers et al., 2024; Rankin et al., 2018; Godman et al., 2014).

Available evidence indicates that the currently available DOACs have broadly comparable efficacy and safety profiles in indications such as non-valvular atrial fibrillation, provided that prescribing recommendations and individual patient characteristics are appropriately considered (Van Ganse et al., 2020; Lip et al., 2024). Consequently, treatment selection may incorporate efficiency criteria without compromising the quality of care, in line with pharmacotherapeutic optimization strategies based on therapeutic equivalence (Godman et al., 2014).

Most patients whose physicians accepted the pharmacist’s recommendation remained on dabigatran throughout follow-up, demonstrating high persistence of the therapeutic switch and successful integration of the optimization strategy into routine clinical practice. Reversion to apixaban or switching to another DOAC occurred infrequently and was primarily driven by individualized clinical considerations, adverse events, or subsequent therapeutic decisions.

Regarding treatment persistence, the high proportion of patients who remained on dabigatran throughout follow-up suggests that the pharmacist-led intervention not only modified initial prescribing patterns but also produced durable therapeutic changes, an important consideration when implementing long-term pharmacotherapeutic optimization strategies. The observed treatment persistence strengthens the estimated economic benefits of the intervention and supports the feasibility of implementing similar strategies in other healthcare settings.

Furthermore, the stability of the overall bleeding rate among anticoagulated patients (5.3% in 2024% vs. 5.2% in 2025) and the absence of a meaningful increase in major bleeding events suggest that the implemented therapeutic changes did not compromise patient safety.

Regarding the observed increase in in-hospital mortality, this finding warrants cautious interpretation: because the complementary and multivariable analyses of hospital-wide mortality were based on administrative ICD-10 coding and the Z79.01 code does not distinguish between VKAs and DOACs, no causal inference regarding the pharmacist-led optimization strategy can be established. These findings should therefore be considered hypothesis-generating and warrant confirmation in studies linking prescription-level data with clinical outcomes (Figures 2, 3).

Overall, the findings suggest that the pharmacotherapeutic optimization strategy was not associated with any evident deterioration in the bleeding safety of the anticoagulated population. The stability of bleeding rates observed following the intervention supports the hypothesis that improvements in prescribing appropriateness and healthcare efficiency can be achieved without compromising clinical outcomes (Table 7).

- Safety and Acceptance of Pharmacists’ Recommendations

Importantly, the observed changes in prescribing practice were not associated with any clinically relevant adverse events during follow-up, further supporting the safety of the intervention.

Although a large proportion of patients were considered candidates for therapeutic optimization, the acceptance rate of pharmacists’ recommendations was moderate, suggesting the persistence of clinical inertia. This phenomenon, widely described in ambulatory care, has been associated with the perceived stability of chronically treated patients and prescribers’ reluctance to modify established therapies, particularly in the absence of overt adverse events (Salahudeen et al., 2022). The variability observed among prescribers further highlights the influence of individual clinical experience and organizational factors on the implementation of pharmacist-led interventions.

One of the most relevant findings, however, was the intervention’s impact on prescribing patterns, particularly among treatment-naïve patients. The marked increase in dabigatran prescribing observed during the second half of the year suggests that the accompanying educational activities and awareness interventions successfully influenced therapeutic decision-making at the time of treatment initiation, a critical stage for achieving sustainable changes in prescribing behavior. This strategy is likely to be more efficient than modifying established long-term treatments and facilitates the adoption of prescribing patterns aligned with both clinical and pharmacoeconomic principles (Rome et al., 2025).

The pharmacist-led intervention not only optimized prescribing but also generated a substantial and measurable economic benefit under real-world clinical practice conditions. Switching appropriate patients to dabigatran, which is associated with a lower annual treatment cost per patient, resulted in a significant reduction in pharmaceutical expenditure without compromising clinical safety. Analysis of prescribing patterns among newly treated patients demonstrated estimated savings of €75,508.81 compared with a counterfactual scenario based on pre-intervention prescribing trends. Additional savings of €31,288.25 were generated through therapeutic switching in 146 patients previously receiving apixaban. Overall, the intervention resulted in total estimated savings of €106,797.06 during 2025, reinforcing its relevance from a healthcare efficiency perspective.

The absence of clinically relevant adverse events during follow-up further supports the safety of this strategy, suggesting that pharmacoeconomically driven optimization interventions can be implemented without compromising patient outcomes (Gorostiza et al., 2025; Korenstra et al., 2016). These findings are consistent with previous studies demonstrating that although DOACs have increased overall expenditure on anticoagulation therapy, their cost-effectiveness can be justified in specific clinical settings, particularly when drug selection incorporates efficiency criteria.

From a population perspective, these findings are particularly relevant in the context of the steadily increasing use of DOACs in Spain. Indicators such as the defined daily dose per 1,000 inhabitants per day (DDD/TID) have shown a sustained increase in DOAC utilization, with the corresponding impact on healthcare expenditure (Agencia Española de Medicamentos y Productos Sanitarios, 2026a). In this context, interventions aimed at optimizing treatment selection at therapy initiation may influence not only individual treatment costs but also overall prescribing trends, thereby contributing to the long-term sustainability of the healthcare system.

The present findings are consistent with previous literature demonstrating that structured pharmacist-led interventions in primary care—particularly those combining individualized medication review, professional education, and feedback to prescribers—improve prescribing quality and reduce inappropriate medication use (Chambers et al., 2024; Salahudeen et al., 2022). Likewise, the multidisciplinary collaboration observed in this study, together with joint educational sessions across healthcare levels, probably contributed to greater consistency in prescribing criteria and higher implementation of pharmacists’ recommendations, in line with multidisciplinary models of pharmacotherapeutic optimization (Godman et al., 2014; Kwint et al., 2011). In this context, the primary care pharmacist emerges as a key contributor not only to the identification of medication-related problems but also as a facilitator of change, educator, and coordinator among healthcare professionals. This multidimensional role supports the transition toward more integrated, patient-centered models of care focused on improving both clinical outcomes and healthcare efficiency.

Although an increase in in-hospital mortality was observed among anticoagulated patients during the post-intervention period, complementary analyses suggest that this finding should not be interpreted as evidence of reduced safety associated with the pharmacist-led recommendations. In a parallel hospital-episode dataset from the same healthcare area (45,424 admissions, 2024–2025), overall in-hospital mortality declined between years (3.61%–3.41%), whereas episodes coded for chronic anticoagulant use (ICD-10 Z^79^.01) showed the opposite trend (age-, sex-, and setting-adjusted year-by-anticoagulation interaction OR 1.89, 95% CI 1.29–2.78; p = 0.001; adjusted OR for 2025 within the anticoagulated subgroup 1.43, 95% CI 1.00–2.03) (Figure 4). This excess mortality reflected a higher per-episode case-fatality rate rather than an increase in admission volume and was not driven by bleeding complications, as haemorrhage-related deaths remained essentially unchanged. Instead, exploratory analyses indicated that excess mortality clustered predominantly among patients with acute kidney injury and cardiorespiratory comorbidity, and the observed association persisted after adjustment for available demographic characteristics and recorded comorbidities, suggesting a residual risk signal not fully explained by coded case-mix. Because anticoagulation status was identified using the administrative ICD-10 code Z79.01, which does not distinguish between VKAs and DOACs, these findings cannot be attributed to the pharmacist-led optimization strategy or to any specific anticoagulant. Rather, they reinforce the importance of structured pharmacotherapeutic surveillance, regular reassessment of renal function and treatment appropriateness, and future studies linking prescription-level data with hospital outcomes, particularly in patients with impaired renal function (Menichelli et al., 2025; Steffel et al., 2021; Bhandari et al., 2025; Tran et al., 2025).

**FIGURE 4:**
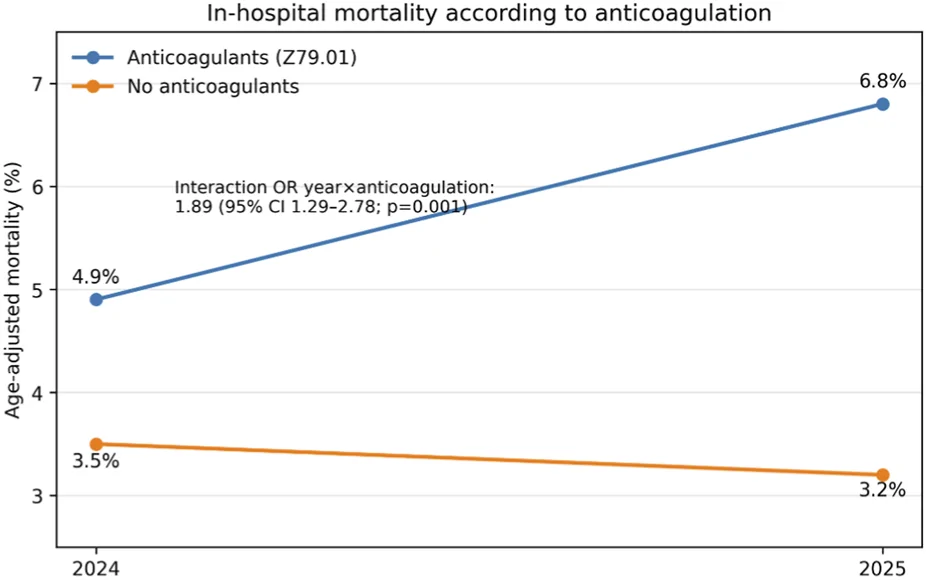
Age-adjusted in-hospital mortality according to anticoagulation status. The anticoagulated subgroup exhibited a reversal of the protective trend observed in the remainder of the cohort.

This study has several limitations inherent to its quasi-experimental design, which preclude establishing a definitive causal relationship between the intervention and the observed changes. Nevertheless, the inclusion of an economic evaluation based on comparative scenarios, together with the large sample size and the real-world clinical setting, strengthens the robustness of the findings and supports their applicability to similar healthcare environments. In addition, the lack of evaluation of hard clinical endpoints and patient-reported outcomes should be considered when interpreting the results.

Overall, these findings reinforce that structured, evidence-based, and multidisciplinary pharmacist-led interventions coordinated across healthcare levels constitute an effective strategy for improving the appropriateness of DOAC prescribing. Such interventions simultaneously enhance patient safety, quality of care, and healthcare system sustainability—three essential priorities in the current context of increasing clinical complexity, population aging, and constrained healthcare resources.

In conclusion, the primary care pharmacist-led intervention promoted the appropriateness of DOAC prescribing through a structured, individualized medication review coordinated across different levels of healthcare. This approach facilitated the identification of patients eligible for therapeutic optimization and promoted a more rational use of DOACs according to each patient’s clinical characteristics and comorbidities.

The intervention also showed a favorable impact on healthcare efficiency by promoting more sustainable prescribing patterns through the selection of clinically equivalent therapeutic alternatives whenever appropriate, particularly among patients initiating anticoagulant therapy.

Furthermore, the structured pharmacotherapeutic review enabled the identification and correction of conditions potentially compromising the safety and effectiveness of anticoagulant therapy, including inappropriate dosing, renal impairment, clinically relevant drug-drug interactions, frailty and patient complexity, and specific prescribing indications.

These findings reinforce the pivotal role of the primary care pharmacist in therapeutic optimization, improving the quality of care, and promoting the efficient and safe use of medicines. Structured, multidisciplinary, and evidence-based interventions seem to represent a valuable strategy for advancing toward more coordinated, safer, and sustainable models of care for anticoagulated patients.

## Data Availability

The datasets generated and/or analyzed during the current study are not publicly available because they contain potentially identifiable clinical and healthcare information and are subject to institutional and data protection restrictions. Requests for access to the datasets should be directed to the corresponding author and will be considered in accordance with applicable ethical, institutional, and data protection requirements.
